# Evaluation of evening versus morning levothyroxine intake in elderly (MONIALE)

**DOI:** 10.1186/s13063-019-3816-3

**Published:** 2019-12-17

**Authors:** Karina Giassi, Vanessa Piccoli, Ticiana da Costa Rodrigues, Renato Gorga Bandeira de Mello

**Affiliations:** 1 Endocrinology Program, Universidade Federal do Rio Grande do Sul, Porto Alegre, Brazil; 2 Internal Medicine and Geriatric Division, Hospital de Clínicas de Porto Alegre, Porto Alegre, Brazil; 3 Endocrine Division, Hospital de Clínicas de Porto Alegre, Rua Ramiro Barcelos, 2350, 4° andar, Porto Alegre, RS CEP 90035-903 Brazil

**Keywords:** Hypothyroidism, Aged, Levothyroxine

## Abstract

**Background:**

The aging population is associated with increased multimorbidity and polypharmacy. Older adults are at a higher risk of adverse events and reduced therapeutic response. This phenomenon is partially explained by drug interactions and treatment adherence. Most randomized clinical trials have found no significant differences between morning and evening levothyroxine (LT_4_) administration in young adults, but there is little evidence regarding alternative LT_4_ regimens in older populations. Thus, the MONIALE trial aims to test an alternative schedule for LT_4_ administration in older adults.

**Methods/design:**

This randomized crossover clinical trial will include participants aged 60 years or older with primary hypothyroidism. The trial groups will consist of morning LT_4_ intake (60 min before breakfast) or evening LT_4_ intake (60 min after supper). The primary outcome will be variation in serum thyrotropin (TSH) levels after 24 weeks of the LT_4_ protocol. The secondary outcomes will be the prevalence of drugs that potentially interact with LT_4_ and hypothyroidism control according to interaction status. The sample size was calculated to detect a minimum mean difference of 1 mUI/L in serum TSH level between the groups with 80% power and a 5% probability of type I error, resulting in 91 patients per group. The project was approved by the Hospital de Clínicas de Porto Alegre Ethics Committee.

**Discussion:**

Considering the aging population, the increased prevalence of multimorbidity and polypharmacy, as well as potential drug interactions and treatment adherence difficulties, an alternative LT_4_ protocol could be useful for hypothyroidism treatment in the elderly. Prior studies comparing alternative LT4 administration protocols have mainly included young adult populations and have not addressed potential drug interactions.

**Trial registration:**

ClinicalTrials.gov, NCT03614988. Registered 30 July 2018.

## Background

Epidemiologic and demographic changes have resulted in aging of the population [1]. Between 1980 and 2017 the number of people aged 60 years or older worldwide has risen from 362 to 982 million, and by 2050 people in this age range will outnumber those in all other age ranges [2].

Older age is associated with a higher prevalence of multiple chronic diseases [3, 4] and polypharmacy, which is generally defined in the literature as the use of five or more concomitant medications [5]. Adverse events, such as drug–drug interactions [6], non-adherence [7], suboptimal therapeutic effectiveness, and poor clinical response [8] are related to multiple drug use. Both multimorbidity and polypharmacy are correlated with falls, hospitalizations, functional limitations, and mortality [9, 10].

The prevalence of thyroid dysfunction increases with age [11, 12]. The National Health and Nutrition Examination Survey, conducted between 1988 and 1994, found a hypothyroidism prevalence of 4.6% (0.3% clinical and 4.3% subclinical), being more common in women aged between 50 and 70 years (*p* < 0.001) [12]. Physiological changes due to the aging process could impact hypothyroidism treatment [13]. In older populations, pharmacokinetics might be modified by gastrointestinal aging and decreases in body water content, serum albumin, hepatic biotransformation, and renal clearance [14].

Levothyroxine is a synthetic derivative (levorotatory isomer) of thyroxine. Its ionization state and dissolution are influenced by gastric pH [15]. Although in healthy volunteers bioavailability can reach 60–80% [16, 17], there could be a 9.4% decrease in thyroxine absorption in patients over 70 years old (62.8% ± 13.5% SD vs 69.3% ± 11.9%; *p* < 0.001), as was found in a study of 45 euthyroid individuals [18]. The small bowel is the main site of absorption; the duodenum accounts for 15 ± 5% SD, the upper jejunoileum 29 ± 14% SD, and the lower jejunoileum 24 ± 11% SD of 24-h ^131^I-labeled thyroxine absorption [16]. The time necessary to reach the maximum serum concentration (Tmax) of the drug is approximately 2–3 h from ingestion, and plateaus occur at 18 and 48 h. Food and hypothyroidism delay Tmax [19, 20].

Drug bioavailability is responsible for most inter- and intra-individual therapeutic variation [6], which can result from (a) nonadherence, (b) physiological (weight, pregnancy, age) and paraphysiological (behavior, nutrition) conditions, (c) malabsorption diseases, and (d) concomitant medications [8].

In an in vitro study, Pabla et al. found that a higher pH impairs dissolution of thyroxine [21], and Centanni et al. observed a higher thyroxine requirement in ten euthyroid patients with multinodular goiter who were receiving concomitant omeprazole [22]. In a prospective study, however, the hormone levels of 19 hypothyroid subjects did not change when they were advised to take omeprazole 30 min after LT_4_ [23]. Besides the known interaction with proton pump inhibitors, interactions between LT_4_ and other drugs/supplements have been recognized [24], such as with iron [25], calcium supplements [26], aluminum hydroxide [27], raloxifene [28], sevelamer [29], cholestyramine [30], and ciprofloxacin [31]. In light of these findings, the American Thyroid Association recommends a 4-h interval between potentially interfering drugs, although this is based on a low grade of evidence [32].

A survey of referral centers about the appropriate use of LT_4_ revealed that, although the majority of patients understood that LT_4_ should not be taken with food, only 52.1% were aware that it should also not be taken with other medications [33]. Forgetting to take the tablets and a lack of understanding about the need for continued treatment were found to be the causes of low adherence in two-thirds of 100 uncontrolled hypothyroidism patients [34].

In 1977, in a sample of healthy volunteers, Wenzel and Kirschsieper found that LT_4_ absorption decreased from 79.3% ± 7.2% SD under fasting conditions to 63.9% ± 10.5% SD after a meal of two buttered rolls and a boiled egg (*p* < 0.001) [17]. Years later, Bevenga et al. demonstrated that postponing breakfast from 15 to 20 min to 60 min improved the thyroid function tests in hypothyroid individuals [20]. LT_4_ has also been reported to interact with coffee [35], soy [36], grapefruit [37], and milk [38].

Although some prospective studies on the timing of LT_4_ administration have been published, they were not designed to include older adults. In a pilot study, Bolk et al. [39] assessed the TSH levels of 19 hypothyroid women (mean age 48 years) for 24 h on two separate days. Evening LT_4_ intake (hours after supper) lowered TSH levels more than morning intake (30 min before breakfast) (1.2 ± 0.3 mUI/L vs 5.1 ± 0.9 mUI/L, *p* < 0.01). Despite the bedtime LT_4_ regimen, the circadian pattern of thyrotropin was maintained and did not interfere with morning blood sampling [39]. The same group was able to reproduce these results in a randomized crossover placebo-controlled trial with 90 hypothyroid patients (mean age 48 years), who were followed for 24 weeks. In the intergroup comparison, bedtime intake had a direct treatment effect, with a decrease in TSH levels of 1.25 mIU/L (95% confidence interval (CI) 0.60–1.89 mIU/L; *p* = 0.001) and an increase in free T4 of 0.07 ng/dL (95% CI 0.02–0.13, *p* = 0.01) compared to intake 30 min before breakfast [40]. Rajput et al. [41] randomized 152 drug-naive hypothyroidism patients (mean age 34.30 ± 11.82 years) into two parallel groups to receive LT_4_ 2 h after dinner or 30 min before breakfast. At the end of 12 weeks, finding no significant difference between treatment strategies for both euthyroidism (96% vs 90%; *p* = 0,19) and mean serum TSH levels (3.27 mUI/L ± 4.19 vs 5.13 mUI/L ± 9.36; *p* = 0.31) [41]. Bach-Huynh et al. evaluated three timing strategies in 65 patients (48 ± 13 years) and found that taking LT_4_ an hour before breakfast resulted in significantly lower TSH levels (1.06 mIU/L 95% CI 0.60–1.52 mIU/L; *p* < 0.001) than when taking it 20 min before breakfast or 2 h after supper [42]. In agreement with these results, Perez et al. found higher serum TSH levels when LT_4_ was taken at the beginning of breakfast (2.89 mIU/L ± 2.82 vs 1.9 mIU/L ± 1.76; *p* = 0.028) than when taken 1 h before [43]. A recent study obtained data from 84 patients (71% aged ≤ 65 years old) on stable doses of LT_4_. They performed a three-period crossover trial of LT_4_ administration (30 min before breakfast, 1 h before lunch and 2 h after supper). No significant differences in thyroid profile results were found in the per-protocol analysis [44].

### Trial rationale

Most published studies on the timing of LT_4_ administration have analyzed young populations and have not addressed the use of intervening medications. This trial aims to test an alternative schedule for LT_4_ administration in older adults with a randomized crossover clinical trial. Morning administration, which is more commonly used, will be compared with evening administration for 3 months of follow-up. This report follows the SPIRIT Statement guidelines [45]. We present a standardized checklist with recommended SPIRIT Statement items (Additional file 1).

## Methods/design

### Study design and setting

This prospective, randomized crossover trial will be conducted at the Endocrinology and Internal Medicine Clinic at the Hospital Clínicas de Porto Alegre, Brazil.

### Eligible participants

The research team will identify outpatients ≥ 60 years old with primary hypothyroidism who have been using LT_4_ for at least 6 months and have been on stable doses for the last 3 months. The study procedures will be explained to those who meet the inclusion criteria, and those who provide written informed consent will be enrolled. Screening will continue until the target population is achieved.

### Exclusion criteria

The exclusion criteria are severe organic syndrome, dementia, thyroid cancer, heart failure (functional class IV), or three or more hospital admissions in the last year due to heart failure decompensation, and refusal to participate.

### Intervention

Participants will be instructed to take LT_4_ tablets according to random treatment allocation, either in the evening (60 min after supper) or in the morning (60 min before breakfast). After 3 months of follow-up, the treatment will be changed using a crossover strategy.

### Outcomes

#### Primary outcome

Change in serum TSH levels after 24 weeks of follow-up.

#### Secondary outcomes

1. To identify concomitant drugs that affect LT_4_ absorption
2. To compare TSH control efficacy between the two treatment strategies according to age, drug interaction, and sex subgroups

### Sample size calculation

SAS Studio 3.7 was used to calculate the sample size. The calculated sample size (182 participants, 91 in each group) can detect a difference of 1.0 mUI/L between the means as significant, considering a standard deviation of 2.0 for group 1 and 3.87 for group 2 (BOLK, 2010). A power of 80% and significance level of 5% were considered in the calculation. Adding 10% for possible losses and refusals, the sample size should be 200.

### Random allocation

A randomization list stratified by sex was created using a web-based program (http://www.randomization.com/). An interchangeable random blocks/variable blocks randomization strategy was chosen to assure allocation concealment. An independent researcher is responsible for the randomization list and treatment allocation.

### Blinding

Due to the crossover design, the trial participants, staff, and outcome assessors will be not blinded, although data analysis will be blinded.

### Data collection methods

**Fig. 1:**
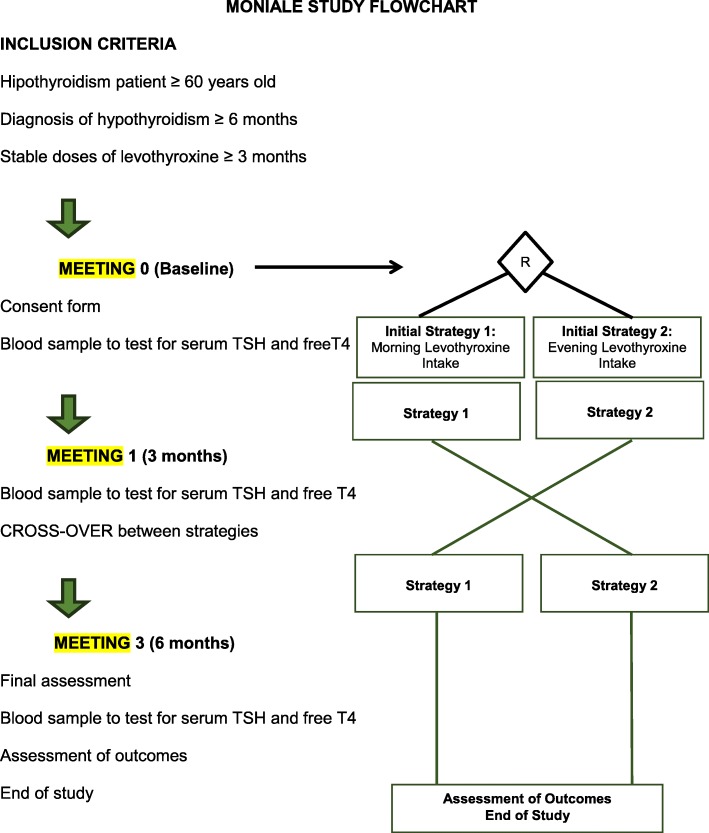
Flow chart of the MONIALE trial: inclusion criteria, randomization, and follow-up process

**Fig. 2:**
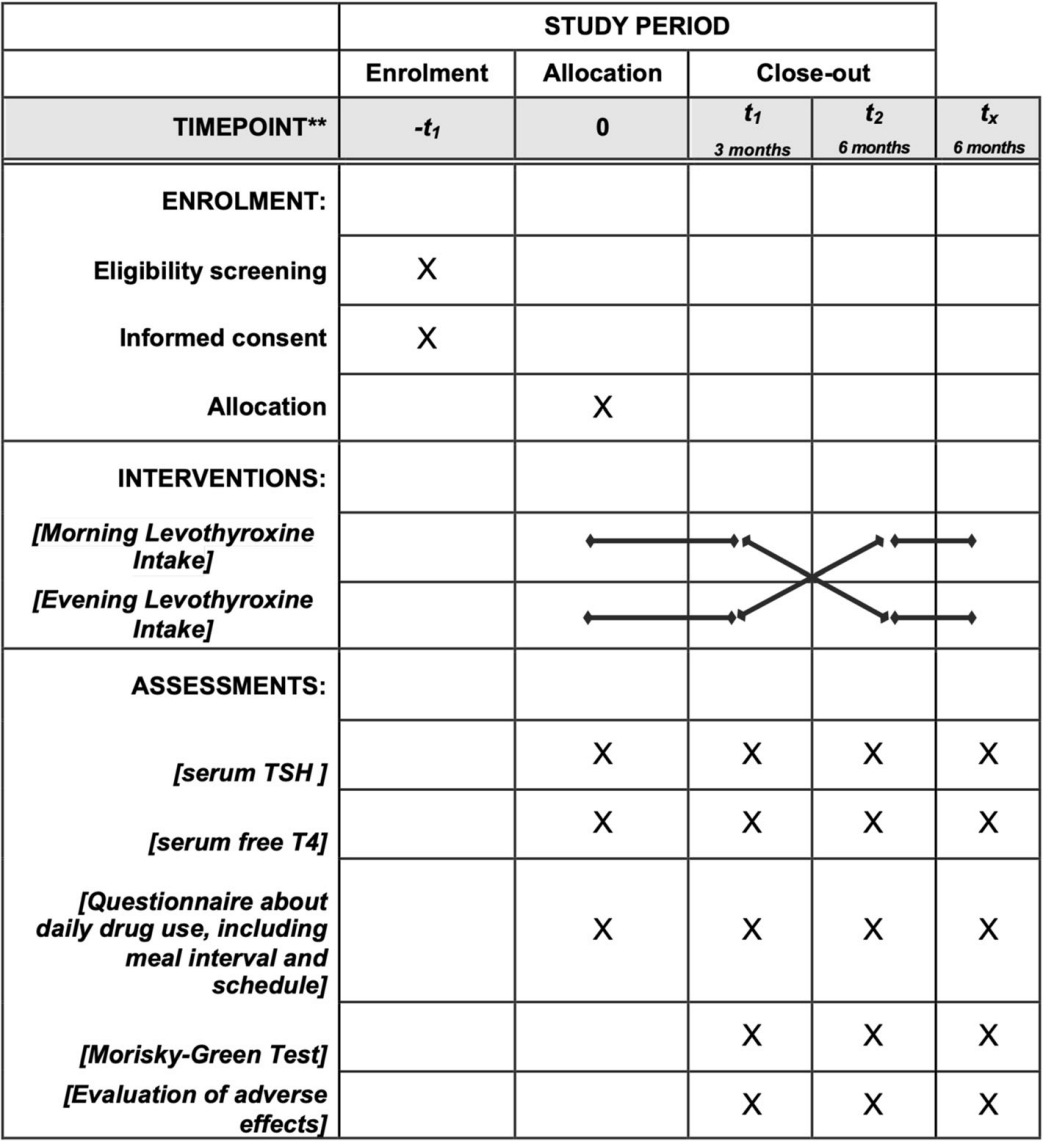
SPIRIT figure: Allocation, interventions and outcomes

Eligible participants will attend meetings at 0, 12, and 24 weeks after enrollment. Each meeting will include application of standardized questionnaires to assess drug–food–thyroxine interactions, treatment adherence, and adverse events. Blood samples will be collected at baseline, 12 weeks, and 24 weeks after initial treatment allocation to measure free T4 and TSH levels. Figures 1 and 2 describe the follow-up procedure.

Thyroid function (serum TSH and free T4 levels) will be measured by electrochemiluminescence assay; concentrations of 0.27–4.2 mUI/L and 0.93–1.7 ng/dL, respectively, are the method’s reference limits. Food–LT_4_ interactions will be analyzed according to time since last meal (< 30 min, 30–60 min, or > 60 min). Concomitant use (within 60 min) of calcium or iron supplements, proton pump inhibitors, other supplements/multivitamins, and daily medications will be considered in the drug–LT_4_ interaction assessment. The Morisky–Green test will be used to verify treatment adherence and comprehension at baseline and follow-up. Side effects will also be investigated regarding causality between LT_4_ treatment and severity. Patients will be discontinued from the allocated intervention upon individual request or worsening of their clinical condition due to decompensated hypothyroidism.

### Statistical methods

To assess the treatment effect, ANOVA for 2 × 2 crossover studies will be performed. The carryover effect will be analyzed by an independent samples *t-*test of the sum of the variables for each patient at 12 and 24 weeks [46]. No interim analysis is planned. Primary and secondary outcomes will be presented as intention-to-treat analysis. Additionally, subgroup analysis comparing age groups of 60–74 years and ≥ 75 years will be performed.

### Data monitoring and auditing

No data monitoring committee will be created due to the single-blind treatment assignment and the short follow-up and interval between thyroid function tests. Although the study coordinator will constantly audit the data and study conduct, no external auditing process is planned.

Logistics procedures are presented in Fig. 1.

## Discussion

Aging is associated with an increased prevalence of multimorbidity (defined as two or more long-term diseases) [4], including a higher frequency of hypothyroidism [12]. Disease-centered, rather than patient-centered, clinical practice guidelines have led to polypharmacy and related adverse events [6, 8]. Due to these issues, in addition to pharmacokinetic changes, hypothyroidism treatment for older adults should be carefully managed [47].

Although alternative strategies of evening LT_4_ administration have been tested in clinical trials [39–44], the mean age of the included patients was under 60 years of age. In addition, most of the published trials have not addressed drug interaction [39–43] as a potential barrier to hypothyroidism control.

This study will evaluate the fastest growing population worldwide and will provide relevant clinical information for their medical care.

## Trial status

Protocol version date 01/19/2018, Institutional Review Board number 2018–0209. This study is currently recruiting participants. The recruitment began in May 2018 and is expected to end by November 2019.

## Supplementary information

**Additional file 1.** SPIRIT 2013 checklist: Recommended items to address in a clinical trial protocol and related documents.

## Data Availability

The applied questionnaires will be stored at the research office with coded identification. The study data will remain unidentified. The final dataset will only be handled by the lead investigator, and the funding institution will not have access to it. The results of this clinical trial will be submitted to a peer-reviewed medical journal following the authorship guidelines of the ICMJE CONSORT Statement. Following study publication, the unidentified dataset will be made available by the corresponding author upon formal request.

## Abbreviations

ICMJE: International Committee of Medical Journal Editors
LT_4_: Levothyroxine
Tmax: Time until maximum serum concentration
TSH: Thyrotropin
